# Wear of the Primary WaveOne single file when shaping vestibular root canals of first maxillary molar

**DOI:** 10.4317/jced.53384

**Published:** 2017-03-01

**Authors:** Daniel Aracena, Eduardo Borie, Pablo Betancourt, Angella Aracena, Mario Guzmán

**Affiliations:** 1 MSc, Dental School, Universidad Mayor, Temuco, Chile; 2 PhD, Research Centre in Dental Sciences (CICO), Dental School, Universidad de La Frontera, Temuco, Chile; 3 MSc, Universidad de La Frontera, Temuco, Chile; 4 Prof, Universidad Autónoma de Chile, Chile; 5 PhD, Mechanical Engineering Department, Universidad de La Frontera, Temuco, Chile

## Abstract

**Background:**

It is very important for a clinician to know the increased wear of mechanized files when establishing endodontic therapy. The aim of this study was to check the wear of the Primary WaveOne file upon shaping two, four and six maxillary molar vestibular canals.

**Material and Methods:**

The deterioration of 40 files, divided into four groups, was evaluated microscopically: group 1, control (unused); group 2, two canals; group 3, four canals; and group 4, six canals. After instrumentation, the files were embedded in resin and sectioned at their apical third into three equal parts. To analyze the wear of edges in the different sections, AutoCAD software was used and analysis of variance (ANOVA) was then performed to compare the mean rake angles.

**Results:**

The files with two and four uses showed slight wear, whereas those with six applications showed significant wear (*p*<0.05).

**Conclusions:**

Primary WaveOne files can be used in up to four root canals without their edges losing effectiveness.

** Key words:**Files wear, reciprocating motion, shaping capacity, WaveOne.

## Introduction

Correct shaping of the canal is essential for the effectiveness of some stages of endodontic treatment, such as chemical disinfection and root filling (1,2).

In recent decades, nickel-titanium (NiTi) alloy, introduced to the field of endodontics in 1988, has become the most widely used memory alloy, as it has a wide range of biomedical applications due to its superelasticity and its capacity to recover its original shape after undergoing great deformations (3,4). With respect to mechanized techniques, this alloy has facilitated the instrumentation of curved root canals with rotational techniques, improving outcome quality in addition to reducing working time and the physical effort of the operator compared with manual techniques (5,6).

Despite the considerable improvements in NiTi instrument design, manufacturing methods and rotary shaping techniques, fractures of files in canals with severe curves still occur (7). Fatigue failure generally occurs due to the formation of microcracks in the surface of the instrument. During each load cycle, microfissures develop from surface irregularities, and these subsequently expand through the material until the instrument completely separates (8). To address this type of accident, optimization of the microstructure of the alloy through new processing technologies has been proposed. Recently, a new NiTi material called M-Wire has been developed through a thermo-mechanical process and shows considerable improvement in resistance to cyclic fatigue compared with conventional NiTi (9-11).

Existing techniques for reciprocating canalicular shaping using a single M-Wire instrument aim to simplify the instrumentation process, to avoid the risk of cross-contamination and to increase resistance to cyclic fatigue (12). One such reciprocating system is the WaveOne (Dentsply-Maillefer®, Ballaigues, Switzerland), whose working angles are 50º clockwise and 170° counterclockwise (13). The advantages of this reciprocating motion are based on the physical laws of action and reaction applied to instrumentation of the canal, which result in a balanced force (14).

At present, there is no available information on the wear that can occur on the single file of the WaveOne system as it is used. The aim of this study was to test in vitro the degree of wear experienced by the Primary file of the WaveOne reciprocating system upon instrumenting 2, 4 and 6 maxillary molar root canals. The null hypothesis was that the primary file of the WaveOne reciprocating system has no significant wear of its edges upon instrumenting two, four and six root canals.

## Material and Methods

This study was approved by the Ethics Committee of the University of La Frontera, Temuco, Chile (DI 16/ 0048).

This study conducted a microscopic evaluation of the deterioration of the edges of 40 Primary single-use 25/0.8 files of the WaveOne reciprocating system (Dentsply-Maillefer®, Ballaigues, Switzerland), which were divided into four groups (n=10 each group). Group 1, or control, corresponded to unused files; group 2 corresponded to files with two instrumented canals; group 3 corresponded to files with four instrumented canals; and group 4 corresponded to files with six instrumented canals.

Between groups 2, 3 and 4, 120 root canals in total were shaped from primary human molars and maxilla extracted from caries and/or periodontal disease. Teeth that were included at random in different groups were selected according to the following criteria: mature apexes, canalicular curvatures equal to or less than 34 degrees, canals with independent foramina, no calcification and no reabsorptions.

The molars were subject to the following protocol: once extracted, they were submerged in a solution of 5.25% sodium hypochlorite (NaOCl) for 20 minutes for disinfection and removal of organic residues. After washing with water, any caries and residual and hydrated restorations were removed from samples, which were then maintained in 2% chlorhexidine solution. Access to the cavity was initially prepared with a high-speed 801L round diamond bur (Jota®, Rüthi, Switzerland) and was then finished by removing all of the chamber roof with an Endozeta bur (Dentsply-Maillefer®, Ballaigues, Switzerland), both cooled with air-water spray. After accessing the canals, the working length was established, subtracting 1 mm of the length of a K 10 file, after visualizing emergence in the apical foramen.

Next, radiographic images were taken of each of the samples with 70 kV equipment (Soredex, Tuusula, Finland) and periapical radiographic film (Ultra Speed, DF58, Kodak®, Rochester, NY, USA) using a work table that allowed standardizing the technique by positioning teeth at a distance of 47.2 cm. The image plates were processed in an automatic developer (Perio Mat Plus, Dürr Dental AG, Bietigheim-Bissingen, Germany), and the images obtained were photographed and processed according to the Schneider technique (15). The AutoCAD 2015 program was used to trace and determine the angulation of root canals. Each of the samples was subsequently mounted in a plaster block to continue with the in vitro instrumentation.

According to the manufacturer’s indication, after scouting the canals with a K 10 file (Dentsply-Maillefer®, Ballaigues, Switzerland), a Primary 25.08 mechanized file of the WaveOne reciprocating system was selected, with which an experienced operator proceeded to instrument the vestibular canals of the sample, using a specific reciprocating motion of 170 counter-clockwise (CCW) and 50 clockwise (CW) and a speed of 350 rpm.

For irrigation, 5% sodium hypochlorite solution was used, and canals were scouted with a K 10 file, with a 2-mm progression into the canal each time the file was applied. An X-Smart Plus motor (Dentsply-Maillefer®, Ballaigues, Switzerland) was used in the shaping of canals with a program indicated for WaveOne system files according to the manufacturer’s instructions.

All of the files used were subjected to sterilization via ethylene oxide to prevent deterioration of the silicone safety installed in the handle.

-Embedding endodontic files

After shaping the respective canals, primary files were embedded inside cylindrical molds filled with vinylester A-430 resin (BASF®, Concón, Chile), which uses cobalt and standard methyl ethyl ketone peroxide as catalysts, both at a ratio of 0.2 ml per 100 ml of resin.

-Sectioning of the files

**Figure 1:**
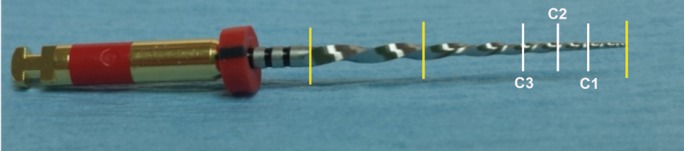
Primary file showing the three sections analyzed (C1, C2 and C3).

The files were sectioned using an EZ lock diamond disc (Dremel®, México DF, Mexico). The apical third of the files was then divided into three equal parts: the first section (C1) was made 1.88 mm from the tip, and after obtaining the corresponding image, the second section (C2) was made at the same distance, to finally make the third section (C3) (Fig. 1). Lastly, the samples were subjected to unidirectional fine grinding for microscopic analysis.

-Measurement of rake angles of the file

A specific platform was built to obtain standardized images of the files. For this procedure, a mobile device was used on which the file was always placed in the same position and at the same distance from the microscope. These images were obtained using a Moticam 5 camera (Motic®, Hong Kong, China) coupled to a stereoscopic microscope (Motic ® model SMZ 168, MoticXiamen, Fujiam, China) with a direct USB connection to a computer. Images were then analyzed with the program AutoCAD® 2015 (Autodesk®, California, CA, USA), determining corresponding cross-sections to the cuts C1, C2 and C3 to then proceeding to measure rake angles in each section after the files shaped zero, two, four and six canals.

-Statistical analysis

Statistical analysis was performed with SPSS v. 16.0 (SPSS Inc., Chicago, IL, USA) with a significance level of 95%. The frequency distribution analysis of the samples (Shapiro-Wilk test) and the homogeneity of variance test (Levene test) revealed that the samples were normal and homogenous, indicating the use of parametric tests. For comparison of rake angles of the files between different sections and uses, analysis of variance (ANOVA) with Bonferroni correction was used.

## Results

The variations of average rake angles of the Primary files in relation to the numbers of uses and sections are summarized in  Table 1.

**Table 1:**
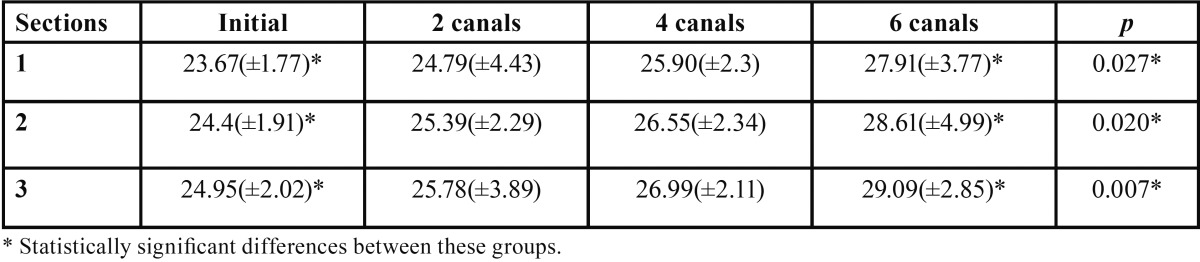
Comparison between the different sections and uses of the files evaluated.

**Figure 2:**
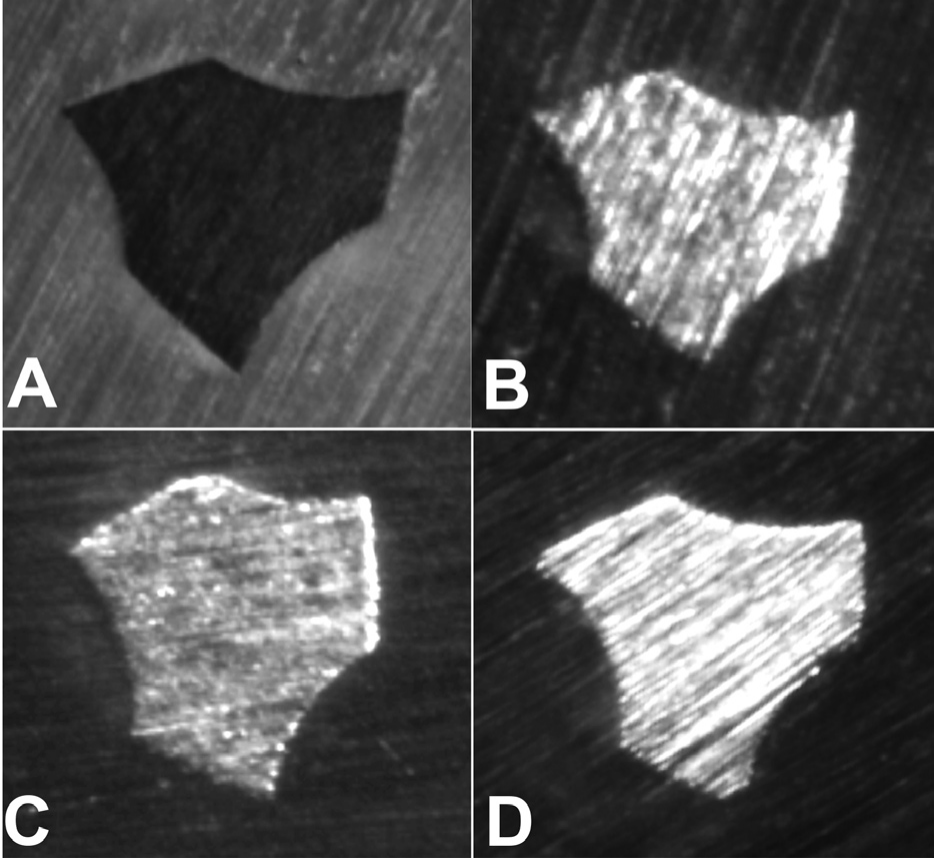
Stereomicroscopic view of the wear of the edges of the Primary files. A) Unused file; B) Used in two canals; C) Used in four canals; D) Used in six canals.

Files used for shaping two, four and six root canals showed negative linear progressive increases in rake angles, causing decreases in the cutting actions of the instrument edges (Fig. 2); however, significant differences were only observed between unused files and files that shaped six canals.

## Discussion

All endodontic files have surface irregularities and internal defects resulting from the manufacturing process, a situation that influences the instrument’s resistance to fracture (16). Accordingly, a large percentage of new Primary files showed various irregularities in their surfaces and different rake angles between each of the samples.

Manufacturers claim that the M-Wire Ni-Ti alloy improves flexibility and fatigue resistance compared to conventional Ni-Ti alloy, which has a high risk of fracture (17,18).

A reciprocal instrument travels a shorter angular distance than a rotary instrument; therefore, it is subject to lower stress values and has higher fatigue resistance (12,13,19), a situation that is corroborated in this study, where of 120 shaped canals, none recorded any separation of the Primary reciprocating file, even when the file was used six consecutive times. Other authors evaluated the deformation and fracture rates of 593 conventional rotary files, reporting 16.02% fractures, confirming the lower resistance of these instruments to cyclic fatigue (7).

Is important to point out that care need to be be taken when using this system in curve canals and with small diameters as reported by Martin *et al.* (20), who stated that a reduction in the angle of curvature may produce a lower incidence of fracture with rotary Ni-Ti instruments. Conversely, Kim *et al.* (21), recommended their use in narrow and calcified canals based on their high resistance to torsion. It is worth mentioning that in addition to root anatomy, other factors contribute to optimal conformation, such as instrument design, instrumentation sequence, file speed, operator experience and use of irrigants (2,22).

Primary single-use files of the WaveOne system proved highly effective for cutting when used in a reciprocal movement. However, the files’ effective cutting action was only maintained through the shaping of four vestibular canals of primary maxillary molars, in contrast to those reported by You *et al.* (13), who studied the behavior of the F2 file of the Protaper system with reciprocal movement in vestibular canals of maxillary and mandibular molars and established their safe use for six canals.

Other researchers that have compared reciprocating systems established that the Reciproc system resisted dynamic and static fatigue cycles more than the WaveOne system and that the probability of half-life was higher for Reciproc than for WaveOne (23,24). Thus, future research should consider extending the study of the wear of files to other mechanized systems.

According to the present in vitro study, the use of Primary files of the WaveOne system can be recommended for the shaping of up to four root canals without the edges losing their effectiveness.
